# Accelerated cardiovascular risk after viral clearance in hepatitis C patients with the NAMPT-rs61330082 TT genotype: An 8-year prospective cohort study

**DOI:** 10.1080/21505594.2020.1870080

**Published:** 2021-01-15

**Authors:** Ming-Ling Chang, Yu-Sheng Lin, Ming-Yu Chang, Chia-Lin Hsu, Rong-Nan Chien, Cathy SJ Fann

**Affiliations:** a Liver Research Center, Division of Hepatology, Department of Gastroenterology and Hepatology, Chang Gung Memorial Hospital, Taoyuan, Taiwan; b Department of Medicine, College of Medicine, Chang Gung University, Taoyuan, Taiwan; c Healthcare Center, Chang Gung Memorial Hospital, Taoyuan, Taiwan; d Department of Cardiology, Chang Gung Memorial Hospital, Taoyuan, Taiwan; e Division of Gastroenterology and Hepatology, Department of Internal Medicine, Chang Gung Memorial Hospital, Yunlin, Taiwan; f Division of Pediatric Neurologic Medicine, Chang Gung Children’s Hospital, Taoyuan, Taiwan; g Institute of Biomedical Sciences, Academia Sinica, Taipei, Taiwan

**Keywords:** HCV, visfatin, pre-B-cell colony-enhancing factor, cardiovascular, NAMPT-rs61330082

## Abstract

Involvement of extracellular nicotinamide phosphoribosyltransferase (eNAMPT, i.e., visfatin or pre-B-cell colony-enhancing factor), a cancer metabokine, in chronically hepatitis C virus (HCV)-infected (CHC) patients with sustained virological responses (SVRs) remains elusive. This 8-year prospective cohort study evaluated eNAMPT profiles of 842 consecutive CHC patients, including 519 who had completed an anti-HCV therapy course and pre-therapy and 24-week post-therapy surveys. For 842 patients, pre-therapy associations were HCV RNA, homeostatic model assessment for insulin resistance (HOMA-IR) index, and body mass index with eNAMPT levels, and NAMPT-rs61330082 T allele with total cholesterol levels. NAMPT-rs10953502, NAMPT-rs2058539, and NAMPT-rs61330082 were in a linkage disequilibrium block, which was associated with total cholesterol levels. Compared to pre-therapy levels, at 24 weeks post-therapy, decreased eNAMPT and increased lipid levels were observed in SVR patients (n = 427). Among SVR patients, higher cumulative incidences of cardiovascular events occurred in those with a NAMPT-rs61330082 TT genotype than those with non-TT genotypes (28.2% vs. 8.4%, *p* < 0.001). NAMPT-rs61330082 TT genotype was independently associated with incident cardiovascular events (95% CI hazard ratio (HR): 1.88–10.37; HR: 4.415); no eNAMPT profiles were associated with incident malignancies. Of CHC patients, hepatic vascular endothelial cells and baseline peripheral leukocytes expressed higher eNAMPT levels than controls, and peripheral eNAMPT-positive leukocyte proportions decreased after SVR. During HCV infection, eNAMPT involvement in glucose metabolism was modulated by HCV RNA linked to lipid metabolism and NAMPT-associated SNPs. Hepatic endothelial cells and peripheral leukocytes potentially secrete eNAMPT. Caution is required for incident cardiovascular events in SVR patients with NAMPT-rs61330082 TT genotype.

## Background

Hepatitis C virus (HCV), classified into eight genotypes [1], is a human pathogen responsible for acute and chronic liver disease that chronically infects an estimated 71.1 million individuals worldwide [2]. In addition to hepatic steatosis, cirrhosis, and hepatocellular carcinoma (HCC), HCV causes mixed cryoglobulinemia [3,4], dyslipidemia, diabetes, obesity, cardiovascular events [5], and extrahepatic malignancies [6]. Although most HCV infections are curable with potent, direct-acting antiviral agents, not all HCV-associated cardio-metabolic complications are reversed after viral clearance [5]. Moreover, HCV-associated malignancies are not eradicable, particularly among patients with baseline diabetes and cirrhosis [7–9]. Thus, identification of a reliable marker and the associated basis for irreversible complications in chronic HCV-infected (CHC) patients after viral clearance may help identify targets for the treatment of these complications.

Nicotinamide phosphoribosyltransferase (NAMPT) is a regulator of the intracellular nicotinamide adenine dinucleotide (NAD) pool [10]. Its gene is located on the 7q22.3 complement strand and consists of 11 exons and 10 introns that span a 34.7-kb region [11]. NAMPT influences the activity of NAD-dependent enzymes via its NAD biosynthesis activity [10], thereby acting as a driver or a pacemaker of metabolism by enhancing cellular proliferation and tipping the balance toward cell survival after a genotoxic insult [12]. Accordingly, NAMPT has been regarded as a molecular link between metabolism and cancer [13]. NAMPT is expressed both intracellularly (iNAMPT) and extracellularly (eNAMPT) [10], with the former functioning in cellular aging and survival and the latter, a highly conserved 52-kDa protein [14], being responsible for transmitting interorgan signals [15]. In addition to its enzymatic function, eNAMPT has cytokine-like activity and is regarded as an adipokine; it is also named pre-B-cell colony-enhancing factor or visfatin [14]. eNAMPT facilitates the switch from innate to adaptive immunity during early inflammation [16], is recognized as a universal marker of chronic inflammation [17], and is directly implicated in regulating glucose-stimulated insulin secretion (GSIS) in pancreatic β cells [10,18]. Moreover, eNAMPT may have cardioprotective properties during ischemia and reperfusion by reducing cardiomyocyte death [16]; however, eNAMPT may also promote vascular inflammation, endothelial dysfunction, and atherosclerosis [17]. Maximal eNAMPT mRNA transcript levels are found in peripheral blood leukocytes and the liver, although the eNAMPT protein is ubiquitously expressed in all tissues [19].

Because both HCV infection and NAMPT influence the host’s immunometabolic functions, their relationship has been investigated but remains elusive. Conflicting results as to whether eNAMPT levels are increased [20] or decreased [21] in CHC patients have been reported. Moreover, the interaction between nutritional homeostasis and eNAMPT is complex; eNAMPT levels are down-regulated by both weight reduction and overnutrition [22] but are up-regulated by nutrient deprivation, caloric restriction, and other forms of mild stress [23]. The situation is even more complicated when the genetic effects of NAMPT are considered. For example, many NAMPT-associated single-nucleotide polymorphisms (SNPs) are associated with special metabolic profiles, such as rs2302559 with serum eNAMPT level [24], rs9770242, and rs1319501 with fasting insulin level, rs9770242 with diabetes-related parameters, rs7789066 with the apolipoprotein B component of very-low-density lipoprotein-cholesterol [25], and rs10487818 with severe obesity [26]. Moreover, the lipid levels have been linked with rs61330082 [27], of which the T allele results in a decrease in the transcription rate [28] and the C-C genotype is associated with high serum eNAMPT and C-reactive protein (CRP) levels [29].

Here, we present the results of an 8-year prospective study that aimed to elucidate the precise role of eNAMPT in HCV infection, with a specific focus on cardiovascular events and malignancies by analyzing the NAMPT profile, including the eNAMPT levels and various NAMPT-associated SNPs, and adjusting for crucial confounders in CHC patients before and after anti-HCV therapy.

## Materials and methods

### Patients

The study group comprised subjects aged 18 years or older with CHC, defined as detectable serum HCV RNA by PCR for >24 weeks. Subjects with human immunodeficiency virus or hepatitis B virus infection, hemochromatosis, primary biliary cholangitis, primary sclerosing cholangitis, autoimmune hepatitis, or malignancy and recipients of solid organ transplants were excluded.

### Study design

A total of 842 patients with CHC were consecutively recruited at a tertiary referral center between January 2010 and June 2017. Of these patients, 519 completed a course of anti-HCV therapy with weight-based pegylated interferon-α-2b and ribavirin for either 24 or 48 weeks [9,30,31]. The HCV RNA levels, HCV genotypes, and interferon-λ3 (IFNL3)-rs12979860 [7,9,30,31] were assessed as previously described. The genotypes of NAMPT-associated SNPs, including rs61330082 [32], rs2302559 [24], rs9770242, rs1319501, rs7789066 [25], rs10953502 [33], rs10487818 [26], and rs2058539 [33], were assessed as described previously [34] (Supplementary Table 1) or were assessed using TaqMan SNP Genotyping assays (Applied Biosystems, Waltham, MA, USA) (Supplementary Table 2). Several baseline factors were recorded, including sex, age, body mass index (BMI), HCV RNA level, HCV genotype, the presence of cirrhosis, and the levels of platelets, the estimated glomerular filtration rate (eGFR), uric acid (UA), total cholesterol (TC), high-density lipoprotein cholesterol (HDL-C), triglycerides (TG), the homeostatic model assessment for insulin resistance (HOMA-IR) [fasting insulin (μU/mL) × fasting glucose (mmol/L)/22.5], C-peptide, high-sensitivity CRP (HS-CRP), alanine transaminase (ALT), and eNAMPT (R&D Systems, MN, USA). For the 519 patients who completed anti-HCV therapy, the aforementioned factors were evaluated 2 weeks before and 24 weeks after completion of therapy. A sustained virological response (SVR) was defined as an undetectable HCV RNA level 24 weeks after completion of therapy. In addition to tracing records of emergency and hospital admissions, patients who achieved an SVR after completion of anti-HCV therapy were followed up every 3 months, and incident cardiovascular events and malignancies were surveyed. The cardiovascular events were defined as ischemic heart disease, coronary revascularization, stroke, heart failure, cardiac arrest, and cardiovascular death identified using the International Classification of Diseases, Ninth Revision, Clinical Modification (ICD-9-CM) codes through patient reports and confirmed by a review of medical records/registries. The malignancies were defined as primary cancers identified using the ICD-9-CM codes. The cancers were diagnosed based on pathology and were confirmed by specialists for each primary cancer; the diagnosis and stage of each cancer were registered with the National Cancer Registration.

A liver biopsy was performed in the CHC patients prior to anti-HCV therapy (n = 20). Control liver samples acquired from the livers of sex- and age-matched normal participants were obtained from the hospital tissue bank (n = 20). Immunohistochemistry (IHC) for eNAMPT (LifeSpan BioSciences, Inc., Seattle, WA, USA) was performed using paraffinized liver samples and peripheral blood (PB) smears according to the manufacturer’s protocols. Smears of isolated peripheral leukocytes were prepared after serial centrifugation of PB [35]. The intensity of protein expression was determined using ImageJ software (http://imagej.nih.gov/ij/, National Institutes of Health, USA).

### Statistics

All statistical analyses were performed using the Statistical Package for Social Science (SPSS package version 21, SPSS Inc., Chicago, IL, USA), Statistical Analysis System (SAS version 9.4, SAS Institute Inc., Cary, NC, USA), PLINK (version 1.07), HAPLOVIEW (version 4.2), or MassARRAY Typer 4.0 (Sequenom) software. Stepwise regression models were used to assess relationships between various dependent and independent variables by adjusting for all independent variables with a *p* value <0.05 (in and out). Paired t-tests were employed to compare variables prior to and 24 weeks after anti-HCV therapy within individuals. Kaplan-Meier estimates and univariate Cox regression were used to assess the relationship of various variables with patient events. Multivariate Cox regression models were used to assess the relationships between various dependent and independent variables by adjusting for all independent variables with a *p* value <0.1 in the univariate analyses. For the genetic analyses, according to our previous studies, population stratification was not indicated [36,37]. SNPs with poor quality were removed using a sequentially exclusive procedure [38,39]. Genotype association tests were performed using logistic regression analyses with the assumption of an additive genetic model. Single-locus association tests were performed in genotype-based, allele-based, and trend-based analyses. Permutation tests based on 100,000 replications were performed to correct for multiple comparisons [40]. Linkage disequilibrium (LD) was computed between every two SNPs to further analyze the haplotype structure [41]. An LD block was determined using the criterion described previously [42]. Within each haplotype block, overall and individual haplotype likelihood-ratio association tests were conducted. Statistical significance was defined at the 5% level based on two-tailed tests of the null hypothesis.

## Results

### Baseline characteristics

The pre-therapy demographics of the CHC patients are listed in Table 1. Compared to the non-SVR patients (n = 92), the SVR patients (n = 427) exhibited lower HCV RNA, HOMA-IR, and C-peptide levels, lower genotype 1 HCV infection and cirrhosis rates, a higher rate of the IFNL3-rs12979860 CC genotype, and higher platelet counts. The pre-therapy eNAMPT levels and the ratios of various NAMPT-associated SNP homozygous genotypes were similar between the SVR and non-SVR patients. Additionally, no difference in the pre-therapy eNAMPT, HS-CRP, and ALT levels was noted among patients with various NAMPT SNP genotypes (Supplementary Table 3).

**Table 1.** Baseline characteristics of the CHC patients

	Total, n = 842	SVR (+), n = 427	SVR (-), n = 92	*p* Values
Male, n (%) #	449 (53.3)	248 (58.1)	47 (51.1)	0.231
Age (year)	55.30±12.50	53.83±11.48	56±10.92	0.086
BMI (kg/m^2^)	24.85±3.875	24.84±3.572	25.54±4.112	0.126
HCV RNA (Log_10_ IU/ml)	4.191±7.338	4.628±8.505	6.220±6.889	<0.001
HCV genotype, n (%)#				
Genotype 1	466 (55.3)	204 (47.8)	74 (80.4)	<0.001
Genotype 2	314 (37.3)	196 (46)	14 (15.2)	<0.001
Others	62 (7.3)	27 (6.3)	4 (4.3)	0.368
Uric acid (mg/dL)	5.91±1.55	5.97±1.55	5.81±1.50	0.404
HOMA-IR	3.18±5.14	2.91±4.41	4.34±5.41	0.028
C-peptide (ng/mL)	2.56±1.92	2.46±1.84	3.35±3.06	0.016
Liver cirrhosis, n (%) #	181 (21.5)	77 (18.1)	36 (39.1)	<0.001
ALT (U/L)	92.21±98.34	100.8±103.8	85.31±78.02	0.188
Platelets count (10^3^/µL)	178.7±66.27	180.9±58.19	154.1±57.68	<0.001
TC (mg/dL)	173.7±34.32	174.3±32.21	173.2±30.24	0.87
TGs (mg/dL)	109.0±59.73	110.4±60.86	100.2±57.90	0.433
HDL-C (mg/dL)	47.71±13.77	47.82±13.89	47.83±14.53	0.993
HS-CRP (mg/dL)	1.93±3.90	1.71±3.56	1.75±3.14	0.913
eNAMPT (ng/mL)	5.93±4.17	5.95±4.06	5.50±3.24	0.382
eGFR (ml/min)	100.5±32.5	100.6±30.6	99.5±33.9	0.779
rs12979860, CC genotype, n (%) #	726 (86.2)	380 (89.41)	65 (70.7)	<0.001
rs61330082, TT genotype, n (%) #	168 (19.9)	96 (22.5)	21 (23.0)	0.477
rs7789066, AA genotype, n (%) #	841 (99.9)	426 (99.8)	92 (100)	0.824
rs10953502, TT genotype, n (%) #	692 (82.2)	349 (80.5)	75 (81.5)	0.889
rs2302559, CC genotype, n (%) #	638 (75.8)	330 (77.3)	64 (70.0)	0.314
rs10487818, AA genotype, n (%) #	842 (100)	427 (100)	92 (100)	NA
rs2058539, AA genotype, n (%) #	653 (77.6)	336 (78.7)	71 (77.4)	0.654
rs1319501, TT genotype, n (%) #	842 (100)	427 (100)	92 (100)	NA
rs9770242, AA genotype, n (%) #	842 (100)	427 (100)	92 (100)	NA

CHC: chronic hepatitis C virus infection; #: chi-squared test; SVR: sustained virological response; BMI: body mass index; Log: logarithmic; HOMA-IR: homeostasis model assessment-estimated insulin resistance; ALT: alanine aminotransferase; TC: total cholesterol; TGs: triglycerides; HDL-C: high-density lipoprotein-cholesterol; HS-CRP: high sensitive C-reactive protein; eGFR: estimated glomerular filtration rate; eNAMPT: extracellular nicotinamide phosphoribosyltransferase; NA: not assessable due to no polymorphism.

### Genetic analyses

**Figure 1.**
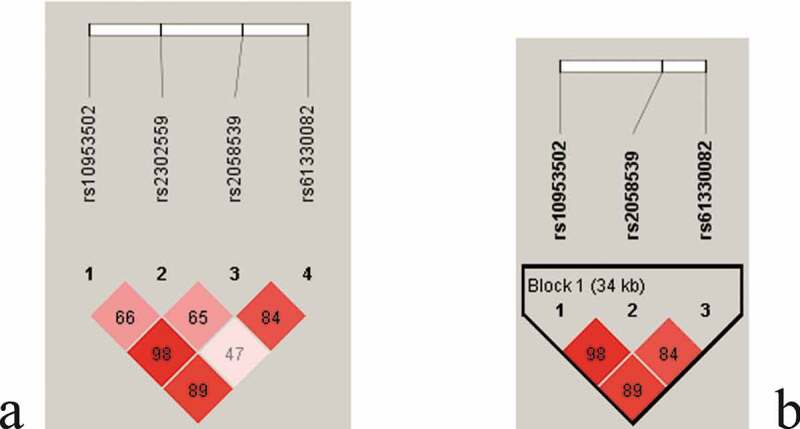
**The linkage disequilibrium (LD) block structure consisted of 4 SNPs (**rs2302559, rs10953502, rs2058539, and rs61330082) (a) and showed a haplotype block constructed by **3 SNPs** (rs10953502, rs2058539, and rs61330082) (b)

Among the nine investigated SNPs (Supplementary Table 2), the genotype distributions of rs12979860 significantly deviated from the Hardy–Weinberg equilibrium (*p* < 0.05) and were deprioritized for the subsequent analysis. The rs10487818, rs1319501, and rs9770242 genotypes were not polymorphic, and the rs7789066 genotype had a minor allele frequency <0.01; these genotypes thus were not analyzed further. Of the remaining four SNPs, rs61330082 was localized in the promoter, rs10953502 was in the intron, and the other two SNPs (rs2302559 and rs2058539) were in the exons of the NAMPT gene. The LD plot constructed using these four SNPs is shown in Figure 1. Figure 1(a) shows the pairwise D’ for the four SNPs. Figure 1(b) shows a haplotype block constructed using the three SNPs (excluding rs2302559). A total of six haplotypes of the rs10953502-rs2058539-rs61330082 block were observed as follows: T-A-T (47.72%), T-A-C (42.33%) C-C-C (9.35%), T-C-T (0.24%), C-C-T (0.24%), and C-A-C (0.12%).

### Pre-therapy associations in CHC patients

**Figure 2.**
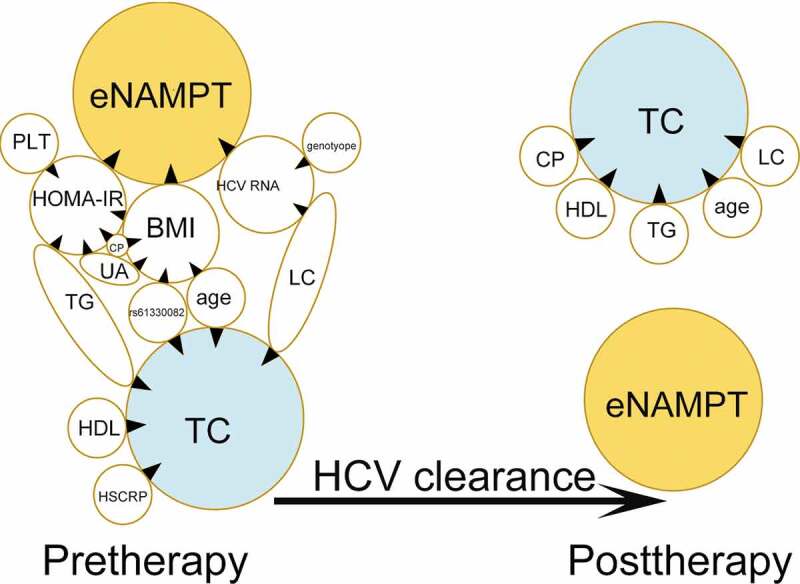
**Cross-sectional eNAMPT- and total cholesterol (TC)-centered associations between dependent and independent factors before (pre-therapy) and 24 weeks after anti-hepatitis C virus (anti-HCV) therapy (post-therapy)**. Tips of black arrowheads: dependent factors; bases of black arrowheads: independent factors; eNAMPT: extracellular nicotinamide phosphoribosyltransferase; HOMA-IR: homeostatic model assessment for insulin resistance; BMI: body mass index; Plt: platelet; G: HCV genotype; CP: C-peptide; UA: uric acid; LC: liver cirrhosis; TG: triglycerides; HDL: high-density lipoprotein cholesterol; HSCRP: high-sensitivity C-reactive protein; SVR: sustained virological response

**Figure 3.**
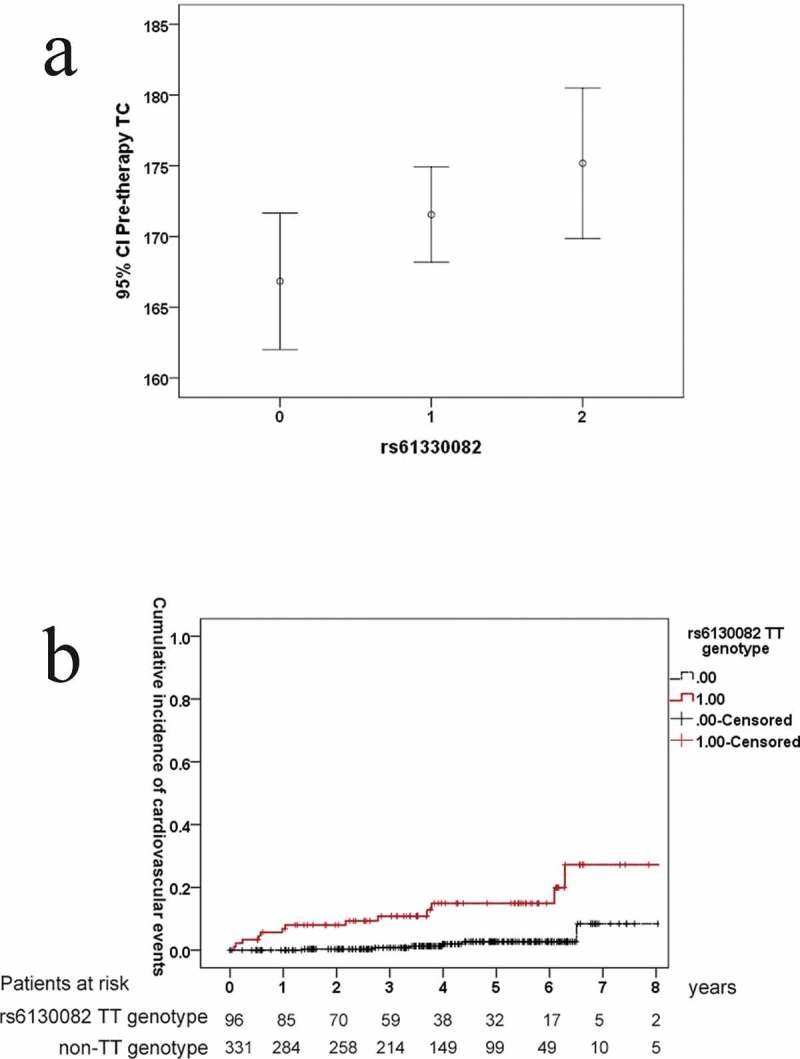
**Pre-therapy total cholesterol (TC) levels among patients with chronic hepatitis C stratified by the NAMPT-rs61330082 genotype**. CI: confidence interval; 0: CC genotype; 1: CT genotype; 2: TT genotype (a) **Cumulative incidences of cardiovascular events in CHC patients with SVRs stratified by the rs61330082 genotype**. Red line: SVR patients carrying a rs61330082 TT genotype; black line: SVR patients carrying a rs61330082 non-TT genotype (b)

For the stepwise regression model, the original variables were pre-therapy factors including sex, age, BMI, HCV RNA, HCV genotype, HS-CRP, UA, eGFR, ALT, TG, TC, HDL-C, HOMA-IR, C-peptide, platelet, liver cirrhosis, rs61330082, rs2302559, rs10953502, and rs2058539, which were used to determine the best model for the pre-therapy eNAMPT levels. HOMA-IR was a positive factor and BMI and HCV RNA were negative factors in predicting the eNAMPT levels in the CHC patients prior to anti-HCV therapy (Table 2, Figure 2). None of the NAMPT-associated SNPs (Supplementary Table 2) were selected in the final model to predict the pre-therapy eNAMPT levels (Table 2). The final model used to predict the pre-therapy TC levels is shown in Table 3. The presence of cirrhosis was negatively associated, whereas age, pre-therapy HS-CRP, HDL-C, TG, and the rs61330082 T allele were positively associated with the pre-therapy TC levels (Table 3, Figure 2). A dose-effect of the T allele of rs61330082 on the TC level was noted, as determined by post hoc tests, the pre-therapy TC levels in patients were as follows: “TT” genotype > “TC” genotype > “CC” genotype (*p* < 0.001) (Figure 3(a)). The T-A-T haplotype of the rs10953502-rs2058539-rs61330082 block was also positively associated with the TC level (Supplementary Table 4). The final models for the pre-therapy HCV RNA, BMI, and HOMA-IR levels are shown in Supplementary Tables 5–7 and Figure 2. As shown in Supplementary Table 6 and Figure 2, the rs61330082 T allele was positively associated with baseline BMI (*p* = 0.034).

**Table 2.** Final model of the stepwise regression analysis for pre-therapy factors associated with pre-therapy eNAMPT levels in CHC patients

Variants	Estimated β	95% CI β	T values	*p* Values
Intercept	11.0591	7.6116 ~ 14.5067	6.29	9.20X10^−10^
BMI (kg/m^2^)	−0.1252	−0.2282~-0.02222	−2.38	0.017718
HCV RNA (Log_10_ IU/ml)	−0.4434	−0.8026~-0.0842	−2.42	0.0160349
HOMA-IR	0.15655	0.0723 ~ 0.2408	3.64	0.0003074

CHC: chronic hepatitis C virus infection; eNAMPT: extracellular nicotinamide phosphoribosyltransferase; CI: confidence interval; BMI: body mass index; Log: logarithmic; HOMA-IR: homeostasis model assessment-estimated insulin resistance.

**Table 3.** Final model of the stepwise regression analysis for pre-therapy factors associated with pre-therapy total cholesterol levels in CHC patients

Variants	Estimated β	95% CI β	*p* Values
Intercept	72.37141	51.32538 ~ 93.41744	6.7X10^−11^
Age (yr)	0.312191	0.048357 ~ 0.576025	0.020967
HS-CRP (mg/dL)	0.88786	0.184982 ~ 1.590738	0.013773
HDL-C (mg/dL)	1.240587	1.009331 ~ 1.471843	1.32 X10^−22^
TGs (mg/dL)	0.231622	0.169855 ~ 0.293389	1.46 X10^−12^
LC (0:no; 1: yes)	−12.655	−19.9286~-5.3814	0.000727
rs61330082 genotype (CC:0; CT:1; TT:2)*	5.195503	1.076845 ~ 9.314161	0.013902

CHC: chronic hepatitis C virus infection; CI: confidence; HS-CRP: high-sensitivity C-reactive protein; HDL-C: high-density lipoprotein cholesterol; TGs: triglycerides; LC: liver cirrhosis; *: additive model.

### Post-therapy associations in SVR patients

For stepwise regression analysis, the original model at 24 weeks post-therapy involved post-therapy factors, including sex, age, BMI, HS-CRP, UA, eGFR, ALT, TG, TC, HDL-C, HOMA-IR, platelet, liver cirrhosis, and the rs61330082, rs2302559, rs10953502, and rs2058539 genotypes; the model showed that none of the investigated variables were associated with the post-therapy eNAMPT level in SVR patients. However, age, post-therapy HDL-C, TG and C-peptide levels, and cirrhosis were associated with post-therapy TC levels (Supplementary Table 8, Figure 2).

### Comparisons between pre- and post-therapy levels of each variable in SVR patients

As shown in Supplementary Table 9, the post-therapy ALT and eNAMPT levels were significantly reduced and the post-therapy lipid levels, including TC, TG, and HDL-C, were significantly increased in the SVR patients. By contrast, none of the aforementioned profiles changed significantly in non-SVR patients.

### Longitudinal follow-up of the outcomes among SVR patients

Our 8-year follow-up evaluations showed that among the investigated NAMPT SNPs, only rs61330082 affected the risk of incident cardiovascular events in the SVR patients, because a higher cumulative incidence of cardiovascular events was noted in those with a rs61330082 TT genotype than in those with a rs61330082 non-TT genotype (28.2% vs. 8.4%, *p* < 0.001) (Figure 3(b)). The cardiovascular events that occurred in the SVR patients included coronary artery disease, stroke, and transient ischemic attack. After adjusting for risk factors of cardiovascular events in multivariate analyses (Table 4), the rs61330082 genotype was still significantly associated with the incident cardiovascular events (95% confidence interval of hazard ratio (HR): 1.88–10.37, HR: 4.415, *p* = 0.001). In contrast, none of the eNAMPT profiles (Supplementary Table 10) were associated with incident malignancies including HCC and lung, skin, gastric, colorectal, esophageal, epiglottic, renal, prostate, and hematologic cancers among the SVR patients.

**Table 4.** Univariate and multivariate analyses of pre-therapy factors associated with incident cardiovascular events among 427 SVR patients

Variants	Univariate analysis: 95% CI of HR (*p* values)	Multivariate analysis: 95% CI of HR [estimated HR] (*p* values)
Sex (male)	0.874 ~ 5.549 (0.094)	0.493 ~ 4.588 [1.509] (0.468)
Age (year)	0.989 ~ 1.062 (0.177)	
BMI (kg/m^2^)	0.926 ~ 1.183 (0.463)	
Smoking (yes)	1.043 ~ 5.354 (0.039)	0.723 ~ 5.706 [2.030] (0.179)
Alcohol (yes)	0.455 ~ 3.277 (0.691)	
Uric acid (mg/dL)	0.792 ~ 1.361 (0.783)	
HOMA-IR	0.946 ~ 1.076 (0.782)	
C-peptide (ng/mL)	0.633 ~ 1.259 (0.893)	
Liver cirrhosis, (yes)	0.635 ~ 3.729 (0.339)	
ALT (U/L)	0.997 ~ 1.005 (0.723)	
Platelets count (10^3^/µL)	0.997 ~ 1.010 (0.345)	
TC (mg/dL)	1.003 ~ 1.027 (0.017)	1.001 ~ 1.029 [1.015] (0.031)
TG (mg/dL)	0.997 ~ 1.021 (0.273)	
HDL (mg/dL)	0.964 ~ 1.025 (0.715)	
HS-CRP (mg/dL)	0.889 ~ 1.15 (0.866)	
eGFR (ml/min)	0.984 ~ 1.016 (0.992)	
eNAMPT (ng/mL)	0.872 ~ 1.128 (0.992)	
rs12979860 (CC:0; CT:1; TT:2)	0.821 ~ 3.849 (0.144)	
rs61330082 (CC:0; CT:1; TT:2)	2.162 ~ 11.305 (<0.001)	1.88 ~ 10.37 [4.415] (0.001)
rs7789066 (GG:0;GA:1;AA:2)	0 ~ 1.089X10^26^ (0.919)	
rs10953502 (TT:0; TC:1; CC:2)	0 ~ 5.553 (0.198)	
rs2302559 (CC:0; CT:1; TT:2)	0.212 ~ 2.293 (0.521)	
rs10487818 (TT:0; TA:1;AA:2)	NA^1^	
rs2058539 (CC:0; CA:1; AA:2)	0.245 ~ 3256.8 (0.168)	
rs1319501 (TT:0; TC:1; CC:2)	NA^2^	
rs9770242 (CC:0; CA:1; AA:2)	NA^3^	

CI: confidence interval; HR: hazard ratio; BMI: body mass index; HOMA-IR: homeostasis model assessment-estimated insulin resistance; ALT: alanine aminotransferase; TC: total cholesterol; TG: triglycerides; HDL-C: high-density lipoprotein-cholesterol; HS-CRP: High sensitive C-reactive protein; eGFR: estimated glomerular filtration rate; eNAMPT: extracellular nicotinamide phosphoribosyltransferase; NA^1^ and NA^3^: not assessable due to all AA genotype; NA^2^: not assessable due to all TT genotype.

### eNAMPT IHC

**Figure 4.**
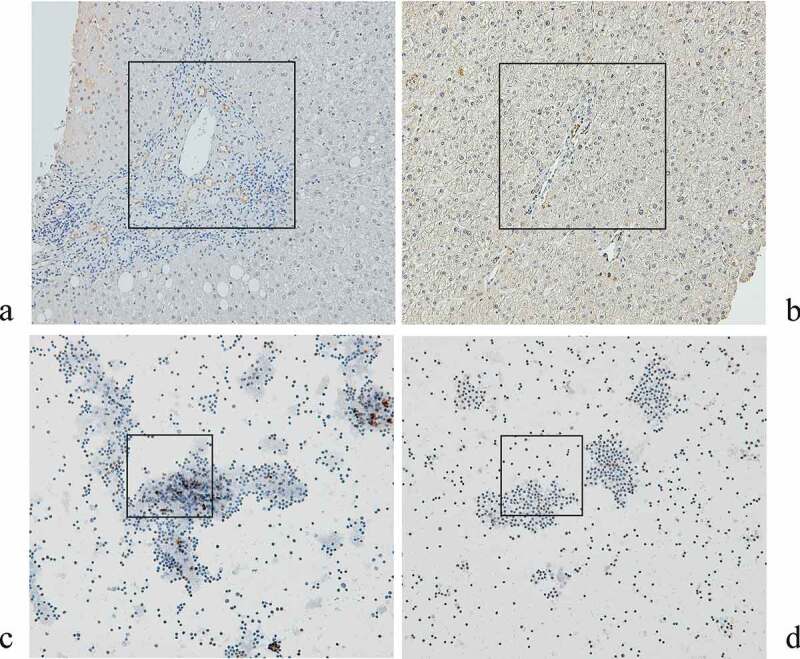
**Immunohistochemistry for eNAMPT in the livers of CHC patients before anti-HCV therapy** (a) **and of control subjects** (b)**, and in PB smears of isolated leukocytes from CHC patients before** (c) **and after** (d) **anti-HCV therapy**. eNAMPT-expressing cells are stained brown. The areas that showed the different density of eNAMPT-expressing cells between (A) and (B), as well as that different between (C) and (D) were marked with black frames

Most of the eNAMPT-positive cells in the paraffinized livers of CHC patients before anti-HCV therapy were vascular endothelial cells (Figure 4(a)). Compared with those of the controls (Figure 4(b)), more eNAMPT-positive cells were noted in the CHC patients (8 ± 2% vs. 0.2 ± 0.1%, *p* = 0.01). Leukocytes comprised all eNAMPT-positive cells in the PB smears of the CHC patients. The proportion of 24-week post-therapy eNAMPT-positive cells decreased (2.64 ± 0.35% vs. 0.67 ± 0.15%, *p* = 0.026) compared to the pre-therapy levels (Figure 4 (c,d)) among the SVR patients. Regarding the non-SVR patients, no significant difference in the number of eNAMPT-positive cells was noted between the pre- and post-therapy PB smears (*p* = 0.382).

## Discussion

All baseline variables that differed between the SVR and non-SVR patients and most of the differences between the pre- and 24-week post-therapy variable values have been consistently reported [5,9,30,31], which strengthened the reliability of the current study’s results. However, the fact that the pre-therapy eNAMPT levels were similar between the SVR and non-SVR patients disqualifies eNAMPT as a predictor for SVR in CHC patients receiving interferon-based therapy. Regarding the associations with eNAMPT, the negative pre-therapy association between the BMI and eNAMPT has been reported [43], and the finding that patients with diabetes mellitus have higher eNAMPT levels than healthy controls [44] is in line with the positive pre-therapy association between the HOMA-IR and eNAMPT. Although obesity and insulin resistance are core components of metabolic syndrome [44], the reverse associations of the baseline BMI and HOMA-IR with the eNAMPT levels suggest that obesity and insulin resistance have opposite impacts on eNAMPT. eNAMPT, also known as visfatin [14], was previously identified as a secreted protein expressed and regulated in adipose tissue through its specific interaction with the insulin receptor, thus exhibiting an insulin-like action; however, these findings have not been reproduced to date [45]. Instead, eNAMPT may be directly involved in the regulation of GSIS in pancreatic β cells [46] through NAD intermediates [10]. In addition, the autocrine effects of eNAMPT are crucial for regulating insulin sensitivity in the liver [47], which is the main target organ of HCV infection. Thus, eNAMPT might counter-regulate insulin resistance through a liver-oriented mechanism negatively modulated by HCV, because the HCV RNA levels were negatively associated with the eNAMPT levels. None of the investigated NAMPT SNPs exhibited any association with the eNAMPT level despite that the rs61330082 TT genotype down-regulated the NAMPT transcription efficiency [28] and rs2302559 was associated with eNAMPT levels [24]; thus, if any associations exist, these investigated SNPs may mainly affect the iNAMPT levels rather than the eNAMPT levels in the case of HCV infection. Moreover, in contrast to previous studies based on French-Canadian [25] and UK [26] populations, rs10487818, rs1319501, and rs9770242 did not meet the criteria for SNPs, which suggested distinct ethnic variance in NAMPT-associated SNPs. However, consistent with the reported connection between the rs61330082 T allele and lipids [27], the rs61330082 T allele was positively associated with the pre-therapy TC level with a dose effect. Furthermore, rs10953502, rs2058539, and rs61330082 were in an LD block and the most frequent haplotype (T-A-T) was positively associated with the TC level, which indicated potentially additive effects of these SNPs in affecting TC of CHC patients.

The finding that only SVR patients had decreased eNAMPT levels 24 weeks after anti-HCV therapy confirmed that HCV clearance led to a decrease in the eNAMPT level. This result seemed to contradict the negative association between the HCV RNA and eNAMPT levels. However, after SVR, all pre-therapy associations with the eNAMPT level, including HCV RNA, HOMA-IR, and BMI, vanished, which suggested an integrated response from the immune and metabolic systems to viral clearance in CHC patients. Of all of the effects of eNAMPT [10,13–17], the proinflammatory characteristic may chiefly explain why the eNAMPT levels decrease after SVR [16,17], at a time when HCV-associated inflammation diminishes. More eNAMPT-positive cells have been noted in the livers (endothelial cells) and peripheral leukocytes of CHC patients than in controls, and most eNAMPT mRNA transcripts are found in the liver and peripheral leukocytes [19]. Moreover, in accordance with the decrease in the serum eNAMPT levels, the proportions of peripheral eNAMPT-positive leukocytes decreased after SVR. Collectively, the immunometabolic impact of eNAMPT in CHC may occur at least partly through *in situ* regulation of hepatic endothelial cells and peripheral leukocytes, both of which are potential sources of serum eNAMPT. We previously reported increased cardiovascular risks in SVR patients at 24 weeks post-therapy, particularly for vulnerable cases with conventional risk factors [48]. Notably, by surveying rs61330082 genotypes, we successfully stratified the SVR patients with risks of incident 8-year cardiovascular events, because patients carrying a TT genotype had a higher risk than their counterparts. Paradoxically, compared with the CC genotype, the TT genotype of rs61330082 is reported to be associated with lower eNAMPT and proinflammatory marker levels in patients with coronary artery disease [44], lower TG but higher HDL-C levels in nondiabetic patients [27], and lower cardiovascular risks in patients with coronary artery calcification [49]. However, no difference in the eNAMPT and proinflammatory marker (including HS-CRP and ALT) levels was found among CHC patients with various rs61330082 genotypes, and the rs61330082 T allele had a positive dose effect on the pre-therapy TC level as mentioned above. These discrepancies may have resulted from differences in metabolic alterations among patients with various diseases and highlighted the homeostatic role of NAMPT. Before viral clearance, HCV-associated hypolipidemia [4,5] may counter-regulate NAMPT SNP-associated hypercholesteremia to achieve metabolic homeostasis, and the cardiovascular risk in CHC patients carrying a rs61330082 TT genotype may be hidden. However, after viral clearance, the reversal of hypocholesteremia seemed to uncover the cardiovascular risk linked with a rs61330082 TT genotype. Moreover, the impact of rs61330082 on the cardiovascular risks existed after adjustment for many pre-therapy factors including TC levels, and rs61330082 was associated with BMI in addition to TC levels. These results suggested that a basis beyond hypercholesteremia might also account for the high cardiovascular risk in SVR patients carrying the TT genotype, which warrants further investigation. Conversely, no difference in the cumulative incidence of malignancies was found between SVR patients with various NAMPT genotypes. Oncogenic pathways other than NAMPT-associated pathways may account for the above dampened effect.

Taken together, our results showed that the involvement of eNAMPT in glucose metabolism during HCV infection was modulated by HCV RNA linked to lipid metabolism and NAMPT SNPs. Hepatic endothelial cells and peripheral leukocytes are potential sources of eNAMPT in CHC patients. After viral clearance, the eNAMPT levels decreased and lipids increased in the CHC patients. For SVR patients, special caution should be taken regarding subsequent cardiovascular events in those with the rs61330082 TT genotype. NAMPT-associated pathways may serve as feasible targets for treating and preventing incident cardiovascular events in these CHC patients after SVR.

## Supplementary Material

Supplemental Material

## Data Availability

The datasets used and/or analyzed during the current study are available from the corresponding author on reasonable request.
