# Supplementary material for: Accelerated cardiovascular risk after viral clearance in hepatitis C patients with the NAMPT-rs61330082 TT genotype: An 8-year prospective cohort study
Source: Virulence. 2021 Jan 15;12(1):270–80. doi: 10.1080/21505594.2020.1870080 (PMC7834047; doi:10.1080/21505594.2020.1870080)
Supplement: Supplemental Material [file KVIR_A_1870080_SM4521.doc]

**Supplementary Table 1. Primer sequences used in the single-nucleotide polymorphisms of NAMPT-rs61330082.**

| **Genes** | **Primers** | **Sequences (5'->3')** |
| --- | --- | --- |
| NAMPT | NAMPT-F | 5′- TGTTTCAAACCTCGTT GCTG -3′ |
|  | NAMPT-R | 5′- GAGGCATGGCTGAGACTTCTA -3′ |

ScrFI for rs61330082—Allele C is cuttable, yielding two fragments of 65 and 138 bp; allele T is uncuttable, and the fragment is still 203 bp.

**Supplementary Table 2. The 9 single-nucleotide polymorphisms (SNPs)** evaluated in the study.

| Gene | SNP ID | ID number of TaqMan assays | Chromosome | Location | Risk allele | MAF | *p*HW^#^ | Beta | 95% CI | *p* value* | Location in relation to  NAMPT |
| --- | --- | --- | --- | --- | --- | --- | --- | --- | --- | --- | --- |
| IFNL3 | rs12979860 | C___7820464_10 | 19q13.2 | 39248147 | T | 0.0870 | 0.000149 | 0.5859 | -0.2757~1.448 | 0.1834 | NA |
| NAMPT | rs61330082 | NA | 7q22.3 | 106248285 | T | 0.4844 | 0.247 | 0.2903 | -0.2686~0.8491 | 0.3093 | promoter |
| NAMPT | rs7789066 | C__29286200_10 | 7q22.3 | 106287306 | G | 0.0019 | 1 | NA | NA | NA | promoter |
| NAMPT | rs10953502 | C__31761492_10 | 7q22.3 | 106251985 | C | 0.0974 | 0.1545 | 0.05404 | -0.8946~1.003 | 0.9112 | intron 10 |
| NAMPT | rs2302559 | C___2673293_10 | 7q22.3 | 106263458 | T | 0.1325 | 0.1524 | -0.1485 | -0.9521~0.6551 | 0.7174 | exon 7 |
| NAMPT | rs10487818 | C___2673297_10 | 7q22.3 | 106269615 | A | 0 | 1 | NA | NA | NA | intron 4 |
| NAMPT | rs2058539 | C__11613113_10 | 7q22.3 | 106276191 | C | 0.1065 | 0.1022 | -0.1096 | -1.028~0.8089 | 0.8152 | exon 11 |
| NAMPT | rs1319501 | C___7590641_30 | 7q22.3 | 106285307 | C | 0 | 1 | NA | NA | NA | promoter |
| NAMPT | rs9770242 | C__30014960_10 | 7q22.3 | 106285885 | C | 0 | 1 | NA | NA | NA | promoter |

IFNL3: interferon λ3; NAMP: Nicotinamide phosphoribosyltransferase; NA: not assessable; MAF: minor allele frequency; ^#^ *P* value of the Chi-square test on the Hardy-Weinberg equilibrium; CI: confidence interval; *The genetic model is an additive model for pre-therapy eNAMPT levels.

**Supplementary Table 3. *P* values (ANOVA) of the comparisons for pre-therapy eNAMPT, HS-CRP and ALT levels among patients with various genotypes (0;1;2) of NAMPT-associated SNPs.**

ANOVA: Analysis of variance; eNAMPT: extracellular Nicotinamide phosphoribosyltransferase; HS-CRP: high sensitivity C-reactive protein; ALT: alanine aminotransferase; SNPs: single nucleotide polymorphisms

|  | eNAMPT | HS-CRP | ALT |
| --- | --- | --- | --- |
| rs61330082 (CC:0; CT:1; TT:2) | 0.304 | 0.878 | 0.188 |
| rs10953502 (TT:0; TC:1; CC:2) | 0.616 | 0.492 | 0.191 |
| rs2302559 (CC:0; CT:1; TT:2) | 0.925 | 0.771 | 0.588 |
| rs2058539 (CC:0; CA:1; AA:2) | 0.635 | 0.763 | 0.313 |

**Supplementary Table 4. The association of haplotype of rs10953502-rs2058539- rs61330082 (T-A-T) with pre-therapy and 24-week post-therapy total** cholesterol levels.

| SNPs | Risk haplotype | Other haplotype | Condition for total cholesterol | MAF | Another allele freq | pHW | beta (OR) | SE | 95% CI of beta | *p* values* | Permutation *p* value (N=100,000) |
| --- | --- | --- | --- | --- | --- | --- | --- | --- | --- | --- | --- |
| rs10953502, rs2058539, rs61330082 | T-A-T | others | Pre-therapy | 0.4772 | 0.5228 | 0.377 | 6.806 | 2.198 | 2.497~11.11 | 0.002094 | 0.00218 |
| rs10953502, rs2058539, rs61330082 | T-A-T | others | Post-therapy | 0.4791 | 0.5209 | 0.3217 | 6.298 | 2.625 | 1.153~11.44 | 0.01687 | 0.01716 |

MAF: minor allele freqquency; pHW: *p* value of hardy weinberg equilibrium test; OR: Odds ratio; SE: standard error; CI: confidence interval; *The genetic model is an additive model.

**Supplementary Table 5. Final model of stepwise regression analysis for pre-therapy factors associated with pre-therapy HCV RNA levels in all 842 patients**.

| Variants | Estimated β | 95% CI β | T values | *p* values |
| --- | --- | --- | --- | --- |
| Intercept | 6.465 | 6.727~6.858 | 65.727 | <0.001 |
| HCV genotype | -0.182 | -0.275~-0.088 | -3.809 | <0.001 |
| Liver cirrhosis (0: no; 1: yes) | -0.284 | -0.543~-0.021 | -0.2115 | 0.032 |

CI: confidence interval

**Supplementary Table 6. Final model of stepwise regression analysis for pre-therapy factors associated with pre-therapy BMI in all 842 patients**.

| Variants | Estimated β | 95% CI β | T values | *p* values |
| --- | --- | --- | --- | --- |
| Intercept |  | 2.850~8.716 | 3.877 | <0.001 |
| eGFR | 0.648 | 0.060~0.081 | 13.420 | <0.001 |
| C-peptide (ng/mL) | 0.319 | 0.414~0.701 | 7.632 | <0.001 |
| Uric acid (mg/dL) | 0.297 | 0.543~0.949 | 7.216 | <0.001 |
| Age (years) | 0.310 | 0.076~0.140 | 6.592 | <0.001 |
| rs61330082 (CC:0; CT:1; TT:2) | 0.086 | 0.038~0.922 | 2.133 | 0.034 |

BMI: body mass index; CI: confidence interval;BMI: body mass index; eGFR: estimated glomerular filtration rate

**Supplementary Table 7. Final model of stepwise regression analysis for pre-therapy factors associated with pre-therapy HOMA-IR in all 842 patients.**

| Variants | Estimated β | 95% CI β | T values | *p* values |
| --- | --- | --- | --- | --- |
| Intercept | -.0492 | -3.922~2.939 | -0.282 | 0.778 |
| C-peptide (ng/mL) | 0.403 | 0.192~0.614 | 3.747 | <0.001 |
| eNAMPT (ng/mL) | 0.199 | 0.089~0.310 | 3.542 | <0.001 |
| Triglycerides (mg/dL) | 0.014 | 0.005~0.023 | 2.978 | 0.003 |
| Platelet (10^3^/µL ) | -0.010 | -0.017~-0.002 | -2.630 | 0.009 |
| BMI (kg/m^2^) | 0.147 | 0.025~0.269 | 2.363 | 0.019 |
| Uric acid (mg/dL) | -0.326 | -0.633~-0.020 | -2.094 | 0.037 |

CI: confidence interval; HOMA-R: homeostatic model assessment for insulin resistance; BMI: body mass index; eNAMPT: extracellular nicotinamide phosphoribosyltransferase.

**Supplementary Table 8. Final model of stepwise regression analysis for post-therapy factors associated with post-therapy total cholesterol levels in 427 SVR patients**.

| Variants | Estimated β | 95% confidence interval of β | *p* values |
| --- | --- | --- | --- |
| Intercept | 71.8629 | 42.88943~100.8364 | 0.020967 |
| Age (years) | 0.84223 | 0.488597~1.195871 | 0.013773 |
| HDL-C (mg/dL) | 0.99532 | 0.652533~1.338109 | 1.32 X10^-22^ |
| TG (mg/dL) | 0.1511 | 0.090684~0.211522 | 1.46 X10^-12^ |
| C-peptide (ng/mL) | 2.63995 | 0.729477~4.550423 | 0.000727 |
| LC (0:no; 1: yes) | -14.928 | -25.397~-4.45908 | 0.013902 |

VR: sustained virological response; HDL-C: high-density lipoprotein cholesterol ; TG: triglycerides; LC: liver cirrhosis

***Supplementary Table 9. Comparison of the pre- and 24-week post-therapy variables in 519 patients underwent anti-HCV therapy stratified by the therapeutic response.***

|  | SVR (+), n=427 | | | SVR (-), n=92 | | |
| --- | --- | --- | --- | --- | --- | --- |
| Variants | Pre-therapy value | Post-therapy value | *p-*values of paired *t*-tests | Pre-therapy value | Post-therapy value | *p-*values of paired *t*-tests |
| ALT (U/L) | 100.8+/-103.8 | 21.83+/-14.82 | <0.001 | 85.31+/-78.02 | 77.71+/-66.89 | 0.463 |
| TC (mg/dL) | 174.3+/-32.21 | 188.0+/-37.75 | <0.001 | 173.15+/-30.24 | 173.2+/-26.43 | 0.406 |
| TG (mg/dL) | 110.4+/-60.86 | 130.3+/-128.6 | <0.001 | 100.2+/-57.90 | 99.26+/-44.81 | 0.169 |
| HDL-C (mg/dL) | 47.82+/-13.89 | 49.44+/-13.40 | 0.001 | 47.83+/-14.53 | 49.18+/-14.22 | 0.286 |
| eNAMPT (ng/mL) | 5.95+/-4.06 | 4.88+/-2.68 | 0.01 | 5.50+/-3.24 | 5.86+/-4.00 | 0.389 |

HCV: hepatitis C virus; SVR: sustained virological response; TC: total cholesterol; TG: triglycerides; HDL-C: high-density lipoprotein-cholesterol ; eNAMPT: extracellular nicotinamide phosphoribosyltransferase.

**Supplementary Table 10. Univariate and multivariate analyses of factors associated with incident oncogenic events among 427** SVR patients.

| Variants | Univariate analysis: 95% CI of HR (*p* values) | Multivariate analysis: 95% CI of HR [estimated HR] (*p* values) |
| --- | --- | --- |
| Sex (male) | 1.166~8.022 (0.023) | 0.608~9.161 [2.36] (0.215) |
| Age (year) | 1.012~1.091 (0.01) | 0.986~1.096 [1.04] (0.154) |
| BMI (kg/m^2^) | 0.833~1.066 (0.342) |  |
| Smoking (yes) | 0.721~3.637 (0.242) |  |
| Alcohol (yes) | 0.959~5.093 (0.063) | 0.8~6.008 [2.192](0.127) |
| Uric acid (mg/dL) | 0.996~1.605 (0.054) | 0.875~1.762 [1.241] (0.226) |
| HOMA-IR | 0.87~1.117 (0.827) |  |
| C-peptide (ng/mL) | 0.8~1.277 (0.574) |  |
| Liver cirrhosis, (yes) | 0.912~4.614 (0.082) | 0.656~4.961 [1.804] (0.253) |
| ALT (U/L) | 0.999~1.015 (0.257) |  |
| Platelets count (10^3^/µL ) | 0.99~1.003 (0.321) |  |
| TC (mg/dL) | 0.986~1.011 (0.841) |  |
| TG (mg/dL) | 0.991~1.008 (0.951) |  |
| HDL (mg/dL) | 0.967~1.025 (0.757) |  |
| HS-CRP (mg/dL) | 0.736~1.167 (0.516) |  |
| eGFR (ml/min) | 0.973 ~1.002 (0.097) | 0.977~1.016 [0.996] (0.709) |
| eNAMPT (ng/mL) | 0.829~1.108 (0.566) |  |
| rs12979860 (CC:0; CT:1; TT:2) | 0.36~2.64 (0.96) |  |
| rs61330082 (CC:0; CT:1; TT:2) | 0.568~1.817 (0.957) |  |
| rs7789066 (GG:0;GA:1;AA:2) | 0~1.1 (0.08) |  |
| rs10953502 (TT:0; TC:1; CC:2) | 0.214~2.394 (0.587) |  |
| rs2302559 (CC:0; CT:1; TT:2) | 0.277~2.218 (0.608) |  |
| rs10487818 (TT:0; TA:1;AA:2) | NA^1^ |  |
| rs2058539 (CC:0; CA:1; AA:2) | 0.322~2.304 (0.766) |  |
| rs1319501 (TT:0; TC:1; CC:2) | NA^2^ |  |
| rs9770242 (CC:0; CA:1; AA:2) | NA^3^ |  |

CI: confidence interval; HR: hazard ratio; BMI: body mass index; HOMA-IR: homeostasis model assessment-estimated insulin resistance; ALT: alanine aminotransferase; TC: total cholesterol; TG: triglycerides; HDL-C: high-density lipoprotein-cholesterol ; HS-CRP: High sensitive C-reactive protein; eGFR: estimated glomerular filtration rate; eNAMPT: extracellular nicotinamide phosphoribosyltransferase; NA^1^ and NA^3^: not assessable due to all AA genotype; NA^2^: not assessable due to all TT genotype.
